# COMPASS Guidelines for Conducting Welfare-Focused Research into Behaviour Modification of Animals

**DOI:** 10.3390/ani16020206

**Published:** 2026-01-09

**Authors:** Paul D. McGreevy, David J. Mellor, Rafael Freire, Kate Fenner, Katrina Merkies, Amanda Warren-Smith, Mette Uldahl, Melissa Starling, Amy Lykins, Andrew McLean, Orla Doherty, Ella Bradshaw-Wiley, Rimini Quinn, Cristina L. Wilkins, Janne Winther Christensen, Bidda Jones, Lisa Ashton, Barbara Padalino, Claire O’ Brien, Caleigh Copelin, Colleen Brady, Cathrynne Henshall

**Affiliations:** 1 Sydney School of Veterinary Science, Faculty of Science, University of Sydney, Sydney, NSW 2006, Australiabidda.jones@sydney.edu.au (B.J.); 2 Animal Welfare Science and Bioethics Centre, School of Veterinary Science, Massey University, Palmerston North 4442, New Zealand; d.j.mellor@massey.ac.nz; 3 School of Agricultural, Environmental and Veterinary Sciences, Charles Sturt University, Wagga Wagga, NSW 2678, Australiaebradshaw-wiley@csu.edu.au (E.B.-W.);; 4 School of Agriculture and Food Sustainability, Faculty of Science, University of Queensland, Brisbane, QLD 4343, Australia; 5 Department of Animal Biosciences, University of Guelph, Guelph, ON N1G 2W1, Canada; kmerkies@uoguelph.ca (K.M.); ccopelin@uoguelph.ca (C.C.); 6 Millthorpe Equine Research Centre, Millthorpe, NSW 2798, Australia; 7 Vejle Equine Practice, Fasanvej 12, 7120 Vejle, Denmark; mette@uldahl.eu; 8 School of Psychology, University of New England, Armidale, NSW 2351, Australia; alykins@une.edu.au; 9 Equitation Science International, Tuerong, VIC 3915, Australia; andrewmclean@esi-education.com; 10 School of Veterinary Medicine, University College Dublin, D04 V1W8 Dublin, Ireland; 11 School of Rural and Environmental Science, University of New England, Armidale, NSW 2351, Australia; 12 Department Animal and Veterinary Sciences, Aarhus University, 8830 Tjele, Denmark; 13 Australian Alliance for Animals, 16 Goodhope Street, Paddington, NSW 2021, Australia; 14 Equine Department, Hartpury University, Gloucester GL19 3BE, UK; 15 Dipartimento di Scienze e Tecnologie Agro-Alimentari, Università di Bologna, 40126 Bologna, Italy; 16 Faculty of Science and Engineering, Southern Cross University, Lismore, NSW 2480, Australia; 17 Institute of Biological, Environmental and Rural Sciences, Aberystwyth University, Penglais Campus, Aberystwyth SY23 3FL, UK; 18 Agricultural Sciences Education and Communication, Purdue University, 915 W State Street, West Lafayette, IN 47907-2054, USA; bradyc@purdue.edu

**Keywords:** conditioning, motivation, deprivation, animal ethics, study design, behaviour modification

## Abstract

Researchers are increasingly engaged in studies to determine and correct negative welfare consequences of animal husbandry and behaviour modification procedures, not least in response to industries’ growing need to maintain their social licence through demonstrable welfare standards that address public expectations. To ensure that welfare recommendations are scientifically credible, the studies must be rigorously designed and conducted, and the data produced must be interpreted with full regard to conceptual, methodological, and experimental design limitations. This commentary provides guidance on these matters. In addition to, and complementary with, the ARRIVE guidelines that deal with animal studies in general, there is a need for additional specific advice on the design of studies directed at procedures that alter behaviour, whether through training, handling, or restraint. The COMPASS Guidelines offer clear direction for conducting welfare-focused behaviour modification research. They stand for the following: Controls and Calibration, emphasising rigorous design, baseline measures, equipment calibration, and replicability; Objectivity and Open data, ensuring transparency, validated tools, and data accessibility; Motivation and Methods, with a focus on learning theory, behavioural science, and evidence-based application of positive reinforcers and aversive stimuli; Precautions and Protocols, embedding the precautionary principle, minimising welfare harms, listing stop criteria, and using real-time monitoring; Animal-centred Assessment, with multimodal welfare evaluation, using physiological, behavioural, functional, and objective indicators; Study ethics and Standards, noting the 3Rs (replacement, reduction, and refinement), welfare endpoints, long-term effects, industry independence, and risk–benefit analysis; and Species-relevance and Scientific rigour, facilitating cross-species applicability with real-world relevance and robust methodology. To describe these guidelines, the current article is organised into seven major sections that outline detailed, point-by-point considerations for ethical and scientifically rigorous design. It concludes with a call for continuous improvement and collaboration. A major purpose is to assist animal ethics committees when considering the design of experiments. It is also anticipated that these Guidelines will assist reviewers and editorial teams in triaging manuscripts that report studies in this context.

## Simple Summary

Negative welfare consequences of the ways animals are managed and used are increasingly being criticised in some sport and production sectors, potentially threatening their social licence to operate. Accordingly, there is a demand for studies aimed at identifying and correcting these welfare compromises by developing standards that more closely align with humane treatment. Preparation for such studies merits planning and guidance. Intended for use in addition to the ARRIVE Guidelines, the new checklist presented here is designed to improve the scientific rigour, competent conduct, and welfare focus of these studies. Presented here are the COMPASS Guidelines, designed to focus attention on the following areas: C—Controls and Calibration; O—Objectivity and Open Data; M—Motivation and Methods; P—Precautions and Protocols; A—Animal-centred Assessment; S—Study ethics and Standards; S—Species-relevance and Scientific rigour. It is expected that they will be useful to scientists, animal ethics committees, regulators, and trainers.

## 1. Introduction

### 1.1. Preamble

Animal Welfare Science, widely acknowledged as a discrete discipline in its own right, currently has well-developed conceptual frameworks that characterise good and bad animal welfare states. It has also identified objectively measurable indices of those states, which are used in the practical management of animal welfare. Its purposes are to promote good welfare in animals with which humans interact. Those animals include, but are not limited to, farm livestock, draught animals, companion animals, wildlife species, some aquatic animals, and animals used for sport, display, or other forms of recreation, such as tourism. In a somewhat different category are animals used in biomedical and veterinary research, teaching, and testing, where the objective is at the very least to minimise any harm to all such animals, especially in cases where the manipulations may be aversive.

The principal conceptual frameworks of animal welfare science today are the products of successive attempts, over the last four decades, to formulate at each stage what animal welfare represents, biologically, and in terms of what animals experience subjectively. These were all framed within the limits of contemporary medical, veterinary, clinical, physiological, affective, and behavioural science understanding of animals [1,2]. As that understanding evolved, so did ideas about what were credible ways to assess animal welfare, and how they could best be represented scientifically. Most early ideas were modified or replaced as the depth and breadth of the salient sciences increased. At each stage, investigations of key features of animal welfare were conducted by conscientiously applying the extant concepts and methodologies. As improvements in understanding are continuous, at intervals, it is important to evaluate which concepts and methodologies need to be discarded, modified, improved, or replaced by new ones. The purpose of the current commentary is to meet this need.

Accordingly, it focuses on factors that can improve the credibility of Animal Welfare Science research at present, as well as the credibility of its practitioners when they seek to make further welfare advances or evaluate the welfare costs of unexamined or newly introduced practices. Highlighted are the fundamental requirements that studies must be rigorously designed and conducted, and that the data produced must be interpreted with full regard to conceptual, methodological, and experimental design limitations.

### 1.2. Replacing Traditional Management Methods with Science-Based Welfare-Focused Practices

The current treatment of animals in production, sport, work, recreation, and display is often shaped by tradition rather than empirical evidence of best welfare practice. In high-turnover production contexts, routine replacement cycles may obscure the welfare costs of short-lived productivity, limiting understanding of their effects on animals and forming patterns increasingly at odds with contemporary societal expectations. These patterns emphasise a critical and urgent need for scientific evaluation of such practices and, where appropriate, to identify and implement effective, improved, and humane substitutes. Provision of such improved standards is essential to stop the habitual overreliance on the remarkable adaptive capacities and behavioural flexibility apparent among many domesticated species, which can lead to unintentional abuse at the hands of poorly informed animal handlers or trainers.

### 1.3. The ARRIVE Guidelines and the Complementary COMPASS Guidelines

The respected, concise, and clearly expressed ARRIVE (Animal Research: Reporting of In Vivo Experiments) Guidelines [3] aim to improve the reporting of animal research studies by ensuring transparency, thoroughness, and reproducibility; they also include ways of promoting best practices in study design, conduct, and reporting. They provide foundational, basic, and generally applicable guidance for all animal-based research studies. Accordingly, readers are strongly advised to read the ARRIVE Guidelines [3] now and before proceeding.

The COMPASS Guidelines, which rely on the generic foundations of the concise ARRIVE Guidelines, focus in more detail on what is required to rigorously conduct research into procedures that alter the behaviour of animals. Their application is restricted in focus to behaviour modification (including restraint practices), and yet is detailed within those limits, by outlining numerous specific elements that are constituents of each guideline.

Accordingly, the COMPASS Guidelines complement the broad foundation of the ARRIVE Guidelines by focusing in more detail on what is required to conduct welfare-focused behaviour modification research. While ARRIVE provides essential standards for conducting and reporting animal research generally, COMPASS addresses the specialised methodological and ethical considerations specific to studies that alter animal behaviour through training, handling, or restraint. The two sets of guidelines serve different but complementary purposes: ARRIVE ensures transparent reporting of what was done; COMPASS provides guidance on how to design and conduct behaviour modification studies rigorously and ethically.

The COMPASS Guidelines include protocols and codes that have been developed to address what are presumed to be unintentional, yet recurring, methodological flaws and welfare concerns identified in contemporary animal welfare science research. The guidelines were formulated to avoid such flaws recognised in earlier reports, several of which were from the present authors. Although some emphasis has been placed on horses and dogs (the two species most often trained), the Guidelines are designed to be generally applicable to studies of animal behaviour modification in a wide range of terrestrial and aquatic animals.

The COMPASS Guidelines include specific points, organised under seven thematic sections, that correspond to the acronym. Their level of detail reflects the broad methodological and ethical considerations involved in behaviour modification research. Readers may find it helpful to consult the PREPARE Guidelines [4], which outline factors associated with formulation of proposed studies, dialogue between scientists and the animal facility, and quality control of components of the study.

### 1.4. Sources of the COMPASS Guidelines

The COMPASS Guidelines are presented as generic principles derived from the direct investigatory experience of the authors involving diverse species and methodologies. Although they are widely used hallmarks of competent research, their inclusion in reports of methodology and related reasoning is usually implicit as opposed to explicit. Accordingly, in this commentary, the COMPASS team members (whose collective wide expertise is acknowledged by their membership of in the team) have, where possible, chosen not to lay claim to specific features of the Guidelines by citing their own work. Instead, they prefer to honour the prior contributions of many others who laid the foundations of the principles that the current team members have applied for some years. It is anticipated that this will contribute to the wider acceptance of the Guidelines and will encourage others to participate in keeping them updated. Having said that, the authors’ direct experience over decades in studies of this sort means their own work is occasionally cited in the Guidelines.

The COMPASS Guidelines were developed through an expert consensus approach involving seasoned researchers with decades of collective experience across diverse species and research contexts. The breadth of these experimental contexts and the uniformity of the principles applied in all of them underpin the credibility of the Guidelines. The development process involved the following: (1) initial conceptualisation by a core group of investigators with extensive direct experience in behaviour modification research and animal welfare assessment; (2) iterative drafting and refinement through detailed interactive review among all co-authors, who collectively bring expertise spanning veterinary medicine, animal behaviour, welfare science, experimental design, statistics, learning theory, industry practice, and regulatory oversight; (3) integration of insights from the authors’ extensive experience of the broadly based research proposals assessed by them while on animal ethics committees and as peer reviewers for leading journals, where they have evaluated hundreds of behaviour modification research proposals and animal-related academic manuscripts; and (4) synthesis of methodological standards that emerged from identifying recurring flaws and welfare concerns in contemporary research.

We acknowledge that this expert consensus methodology differs from the formal Delphi exercise and structured road-testing employed in ARRIVE 2.0 development. However, the principles articulated in the COMPASS Guidelines have undergone extensive informal validation through the authors’ practical application across diverse research contexts. In their roles as peer reviewers and ethics committee members, the authors have applied these standards when evaluating research proposals and manuscripts—identifying methodological shortcomings, requesting additional controls, questioning welfare assessment approaches, and recommending study design improvements. This iterative process across hundreds of proposals and publications, spanning diverse species and research contexts, has refined understanding of what constitutes rigorous, ethical behaviour modification research. The gaps and concerns that the Guidelines address emerged directly from the practical experience of what has been consistently missing or was inadequately addressed in submitted work.

### 1.5. Research Impact and Implementation

The development of COMPASS Guidelines addresses critical gaps encountered by animal ethics committees, regulatory bodies, and research institutions seeking evidence-based standards for evaluating behaviour modification research proposals and publications. By providing explicit criteria for study design, data collection, and welfare assessment in this specialised field, the Guidelines enable more consistent peer-review processes and facilitate meta-analyses by standardising reporting approaches across institutions and species. The guidelines support researchers in demonstrating scientific rigour to funding bodies while ensuring that welfare recommendations emerging from such studies carry sufficient credibility to inform industry practices and regulatory decisions. As an evolving framework, the COMPASS Guidelines are designed to be updated as new methodologies and welfare assessment tools are validated, ensuring that behaviour modification research continues to meet the highest standards of scientific and ethical practice.

We welcome structured feedback from researchers, animal ethics committees, and journal editors who apply these guidelines in evaluating behaviour modification research, proposals, and manuscripts. As the Guidelines are designed as an evolving framework, systematic documentation of their application across diverse research contexts will inform future refinement and validation of these standards. We encourage the research community to share their experiences with COMPASS implementation, whether through correspondence, published commentaries, or formal validation studies, to ensure these Guidelines continue to meet the highest standards of scientific and ethical practice.

## 2. The COMPASS Guidelines

Each set of guidelines is identified by its principal letter with subheadings indicating key areas within each set. Guidance in each case is provided discursively or as bullet points. Repetition occurs only where a similar guideline applies to a different feature under another heading. For orientation, the following sections detail the 124 points that comprise the COMPASS Guidelines: 23 under Controls and Calibration, 20 under Objectivity and Open Data, 30 under Motivation and Methods, 9 under Precautions and Protocols, 16 under Animal-centred Assessments, 9 under Study ethics and Standards, and 17 under Species-relevance and Scientific rigour. To improve clarity, each point is configured as a brief purpose identifier followed by one or more direct statements about how the identified purpose can be achieved. Additional explanation is provided where deemed to be helpful.

### 2.1. C—Controls and Calibration

These attributes of experimental design are essential for the interpretation of results from studies of behaviour modification, training, handling, and equipment use to be credible. They highlight the need for rigorous design, baseline measures, equipment calibration, and replicability.

#### 2.1.1. Subject Selection and Characterisation

- Scientific and ethical balance: Subject selection directly affects both the validity of experimental results and the replicability of studies. The choice between naïve and experienced animals has implications for experimental control, while characterisation of subjects enables future researchers to assemble comparable cohorts. These decisions must balance methodological rigour with ethical responsibility, recognising that compromised experimental design may itself constitute an ethical failure if it results in animals being exposed to procedures that yield unreliable data.
- Naïve versus experienced animals: There is merit in recruiting subjects who are naïve to treatments or procedures, and who are not habituated to handling by researchers. Experienced animals have a more variable learning history than naïve animals, which makes it difficult to replicate studies that have used them. The use of naïve subjects thus provides stronger experimental control and clearer interpretation of results.
- Use of naïve animals in aversive studies: In studies involving aversive stimuli or restrictive equipment, balance the scientific advantages of prioritising the use of naïve animals over those habituated to the intervention against the cost of exposing individuals to aversive stimuli. Where naïve subjects are recruited, animal guardians must be fully informed of all potential risks at the point of recruitment.
- Use of habituated animals: Clearly justify the use of habituated animals and acknowledge associated limitations when interpreting the results. Alternatively, both experienced and naïve animals may be used as a control group.
- Characterisation of experience: Describe the experience level of animals using validated instruments, e.g., Canine Behaviour Assessment and Research Questionnaire (C-BARQ) [5] for dogs, or Equine Behaviour Assessment and Research Questionnaire (E-BARQ) [6] for horses. The purpose is to facilitate replication and identification of comparable cohorts for future studies.

#### 2.1.2. Baseline Measurements

- Baseline scope: Collect baseline data for all relevant welfare indicators, including physiological and behavioural measures. For physiological indicators, record a minimum of 15 min [7]. Infrequent or variable indicators, such as those subject to repeated diurnal variations, may justify longer baseline data collection periods.
- Individual variation: Select animals appropriate to the study and establish individual baselines rather than relying solely on group means.
- Washout periods: Implement adequate washout intervals between treatments to ensure independence of measurements. Report evidence that the duration chosen is appropriate for the measured variables and species studied. Preferably, use contemporaneous control animals to determine the required washout duration.

#### 2.1.3. Pressure, Tension, and Force Measurements

- Calibration: Use sensors validated for the species being studied, and calibrate pressure, tension, and force measurement equipment before and after the study, specifying the calibration method.
- Sensor placement: Describe anatomical locations of sensor placement precisely in relation to standardised landmarks.
- Peak and mean values: Report both peak and mean pressure values, as peak pressures often provide the most relevant welfare information.
- Minimum values: Include minimum values to capture potential low thresholds of pressure or force.
- Spatial distribution: Present pressure distribution patterns in addition to point measurements.

#### 2.1.4. Behavioural Assessment

High-quality behavioural assessment of scientific value depends on systematic methods, often involving repeated analysis of video recordings to capture various behaviours.

- Ethogram development: Develop comprehensive, validated ethograms specific to the study context. Each behavioural element must be mutually exclusive, clearly defined, and useable by independent observers. Avoid circular descriptions (i.e., avoid using the term being defined within the definition itself).
- Visual documentation: Whenever possible, include high-definition photographic or video examples of recorded behaviours.
- Indicators of negative affect: Prioritise documentation of behaviours associated with pain, fear, distress, and/or animals’ attempts to resolve or avoid noxious stimuli. Consider whether equipment, restraint, environmental constraints, or other factors may inhibit or distort behavioural expression, potentially confounding welfare assessment and study outcomes.
- Indicators of positive affect: Recognise and document behaviours potentially indicative of positive experiences, where their interpretation is appropriate for the species and supported by critical evaluation.
- Objective terminology: Exclude subjective and anthropomorphic descriptors (e.g., “intense stare,” “glazed expression”) that cannot be objectively verified by independent observers.
- Observer reliability: It is preferable to avoid single observers. Conduct behaviour coding (from videos) by observers who are competent with the ethogram and who, as far as is practicable, are blinded to treatment. Report inter- and intra-observer reliability analyses, with at least 10% of data reviewed. Target inter-observer (different observers) reliability correlation coefficients (Pearson’s r) of ≥0.80 and intra-observer reliability (same observer across time) generally should show correlations above 0.85.

#### 2.1.5. Physiological Measurements

- Validation requirements: Use only validated physiological indicators with demonstrated reliability in the species being studied. This validation may be derived from other peer-reviewed studies that used the method or against behaviour measures.
- Contextual relevance: Ensure that the selected physiological measures are appropriate for the specific context of the study. Consider whether they reflect the type of physiological response expected under the conditions being investigated, and whether they may be confounded by other factors (e.g., habituation, physical activity, environmental conditions).
- Individual baselines: Establish individual baseline values rather than relying solely on population norms.
- Measurement duration: Conduct pilot studies based on peer-reviewed published research to ensure measurement periods will detect meaningful changes.

### 2.2. O—Objectivity and Open Data

Objectivity with validated tools and open data will ensure transparency.

#### 2.2.1. Statistical Analytics Specific to Behaviour Modification

- Biological significance: It is preferable to report on the biological (adaptive) rather than solely the statistical significance of findings. When discussing results, adopt an and biological significance, gauged by whether the latter would matter to the animals. Biological significance tells us whether the reported difference matters in the real world; focusing on this encourages researchers to address whether the effect is large enough to impact, for example, survival, reproduction, or ecosystem function.
- Contextualisation: Consider results within the existing literature rather than focusing solely on the perceived significance of *p*-values [8]. Additionally, report on animals that show individual differences of concern that may be overlooked if an intervention has no overall mean effect. Always include reasons for removing individual animals from statistical analyses or the study.
- Sample size and power: The number of animals included in a welfare study directly influences the reliability, statistical power, and generalisability of the findings. There may be species-specific challenges associated with statistical sample size when researching groups of horses or dogs that display greater genetic diversity compared to, for example, line-bred mice. With an emphasis on the biological importance rather than the statistical significance of results, it is anticipated that studies adhering to the COMPASS Guidelines could add to the bank of candidate studies for meta-analysis, thereby increasing overall sample size.
- Standard deviations: Present standard deviations to facilitate calculation of optimum sample size for future studies [9]. An insufficient sample size may compromise the reliability and statistical validity of study results, whereas an excessively large sample may raise ethical concerns. On the other hand, the use of data from individual animals can be informative [10,11]. Determining an appropriate number of animals through careful calculation is essential to ensure that the study is both scientifically robust and ethically justified (see ARRIVE guidelines).
- Group-level replication: When there is a risk of social facilitation of behaviours within groups, the group, rather than the individual animal, should be treated as the experimental replicate for those behaviours. Reports should include the number of excluded animals and the reasons for their exclusion.

#### 2.2.2. Conflicts of Interest and Industry Relations

##### 2.2.2.1. Industry Collaboration

- Welfare priority: Ensure that animal welfare considerations take priority over commercial, performance, or competitive interests.
- Appropriate collaboration: Only accept as appropriate collaboration with industry if scientific independence in experimental design and delivery is assured.
- Transparent partnerships: Clearly define roles and responsibilities in industry partnerships.

##### 2.2.2.2. Financial Disclosures

- External funding: Clearly disclose any funding or financial relationships to all of those involved in the research (including animal owners) and in the publication of the findings. Confirm that neither funding bodies nor their agents were involved in experimental design, interventions, or interpretation of results.
- Equipment provision: Disclose any equipment or materials provided by commercial entities.
- Consulting relationships: Report any consulting or advisory relationships relevant to the research.
- Patent interests: Disclose any patent applications or intellectual property interests.

##### 2.2.2.3. Study Independence

- Protocol independence: Ensure research protocols are developed independently of commercial, sporting, or competitive interests.
- Data analysis independence: Maintain independence from funding bodies in data analysis and interpretation.
- Independent publication rights: Maintain independence from funding bodies in writing reports and retain rights to publish findings regardless of outcomes.
- Peer review: Subject all findings to independent peer review before publication, ensuring that all referees are free of conflicts of interest that would affect their objectivity.

##### 2.2.2.4. Data Availability

- Open data: Make de-identified (anonymised) data collected during protocols available via data repositories.
- Integration of study design: Incorporate open-data principles into experimental design and include consent procedures during human and animal subject recruitment.
- Participant anonymity: Design the collection of potentially identifying materials (e.g., video, still photography) to minimise identification while allowing for meaningful deposition into data repositories.
- Informed consent: Where appropriate, obtain broad informed consent to permit use, analysis, and distribution of data as outlined in the study protocol.

### 2.3. M—Motivation and Methods

The animals’ motivation to interact with the experimental environment must be clearly understood, and studies should use standardised equipment and methods with a focus on learning theory, behavioural science, and evidence-based use of positive reinforcers and aversive stimuli. In addition, it is important to recognise the role of subjective emotional affect and intrinsic motivation within animal–human interactions [11,12,13].

#### 2.3.1. Equipment and Intervention Studies

##### 2.3.1.1. Equipment Design and Fitting

- Standardised fitting: Develop and report standardised fitting procedures for all equipment, including, for example, in horses, curb chains.
- Stipulate ‘goodness-of-fit’ criteria: provide specific repeatable indices for grading how well different types of equipment fit, scaled against welfare impairments and benefits.
- Individual ‘fit’ assessment: Report the assessment protocol for fitting of equipment on each animal.
- Standardise equipment use within groups: Notwithstanding individual fit assessments, in studies of gear such as bits or nosebands, the equipment should otherwise be identical within study groups.
- Alternative configurations of equipment: Test relevant alternative configurations if appropriate for the study in question (e.g., for horses: different noseband types, bit arrangements).
- Wear duration: Study equipment in use for durations that align with real-world training and competition settings, not just brief testing periods.

##### 2.3.1.2. Pressure and Restriction Studies

- Graduated testing: Use graduated pressure/restriction levels with clear endpoint criteria for animal welfare.
- Multiple measurement locations: Where relevant, assess pressure at multiple relevant anatomical locations.
- Soft tissue considerations: Account for differences in underlying soft or hard tissue when interpreting pressure measurements.
- Movement artefacts: Control for or account for movement-related artefacts in pressure measurements (e.g., data collected in one gait may differ from those collected in another or in the stationary animal).

##### 2.3.1.3. Training Method Comparisons

- Matched prior training experience: Use validated instruments to ensure animals in different training groups have similar overall training experience when assigning them to treatment groups to assemble similar cohorts.
- Trainer standardisation: Use standardised training protocols to report influences on both behaviour and affective state. Use multiple trainers to reduce trainer-specific effects. To ensure that operator cues are consistent in treatment and control groups, the use of objective tools, such as rein tensiometers, should be considered within the study design.
- Blinding: Personnel participating in the study should, as far as possible, be unaware of the expected outcomes of the study to avoid unintentional cueing (the *Clever Hans* effect).

#### 2.3.2. Specific Guidelines for Common Types of Animal Training Studies

##### 2.3.2.1. Equipment Studies

- Anatomical considerations: Account for anatomical differences in underlying tissues at measurement sites.
- Functional assessment: Include assessment of oral function (chewing, swallowing) under realistic and repeatable conditions.
- Pressure distribution: Assess pressure distribution patterns, not just point measurements.
- Temporal factors: Study effects over realistic periods, not just brief exposures.

##### 2.3.2.2. Studies of Training Methods

- Law and ethics: Ensure compliance with local legislation and provide animals with appropriate warnings (e.g., auditory cues) before administering any aversive stimuli.
- Learning theory application: Ensure training methods are analysed using established learning theory and the generally applicable International Society for Equitation Science (ISES) Principles of Training [14].
- Welfare-based outcomes: Include welfare-based outcome analyses (using the 2020 Five Domains Model) alongside performance measures [15].
- Long-term assessment: Include follow-up assessments where feasible to evaluate long-term effects.
- Stress assessment: Include a comprehensive stress assessment of animal participants during training sessions using multiple behavioural and physiological indicators.
- Distinguish pain from conflict behaviours: Recognise that many behaviours may arise from conflicting motivations due to simultaneous or contradictory anthropogenic signals rather than musculoskeletal pain. Assessment protocols should systematically rule out pain before attributing such behaviours to training-related causes.

##### 2.3.2.3. Equipment Evaluation Studies

- Real-world testing: Test equipment and the effect of training methods under realistic use conditions.
- Comparative design: Ensure that studies of equipment include relevant and nuanced comparison protocols, not just outcomes with and without the focal equipment.
- When aiming to investigate whether existing guidelines are appropriate, ensure inclusion of treatments in both directions (e.g., with horses, both tighter and looser nosebands).
- Individual fitting assessment: Assess equipment fit and adjustment for each animal.
- Standardised equipment use within groups: Notwithstanding individual fit assessments, equipment such as bits, padding, or nosebands should otherwise be identical within groups.
- Wear duration: Study equipment effects over realistic wear periods.
- Movement analysis: Movement analysis should assess the effects on natural movement patterns, including non-fixed body postures as well as fixed discipline-specific frames (such as hyperflexed frames, e.g., in dressage studies).

### 2.4. P—Precautions and Protocols

Precautions and protocols must embed the precautionary principle, stop criteria, and real-time monitoring.

#### 2.4.1. Animal-Centred Welfare Assessment

- Precautionary principle: When evidence is uncertain or conflicting, err on the side of caution to avoid potential harm to animals.
- Five Domains: Use the Five Domains approach [15] to assess consequences of the intervention on animals.
- Subjective experience priority: Welfare assessment must prioritise supportable inferences of the animal’s subjective experience over convenience-based measurements.
- Comprehensive evaluation: Welfare cannot be measured by single indicators but requires a comprehensive assessment of multiple physiological and behavioural factors, interpreted with regard to the animals’ evolved capacities and individual variation, situational context, biological state, and learning history.

#### 2.4.2. Scientific Integrity

- Evidence-based conclusions: Draw conclusions that are directly supported by, and limited to, the data presented, avoiding extrapolation beyond the scope of findings.
- Research conduct: Follow recognised codes of responsible research conduct.

#### 2.4.3. Responsibility

- Animal-centric perspective: Research design and conclusions must balance what is measurable with what is meaningful to the animal, ensuring that study design and interpretation reflect what matters to them in welfare terms, rather than what is merely convenient for human measurement.
- Protection from harm: Research protocols must actively mitigate stress, pain, fear, and other adverse effects on participating animals, including any lasting or cumulative effects that may compromise their future welfare. Particular care is required when procedures involve known or credibly suspected risks, where precaution must take precedence.
- Broader implications: Researchers should consider the wider consequences of their work for animal welfare across contexts, for public trust in science, and for the ethical standards of the disciplines involved.

### 2.5. A—Animal-Centred Assessments

Animal-centred assessments should feature multimodal welfare evaluation, using physiological, behavioural, and functional indicators that are biologically meaningful in light of the animal’s evolved capacities and individual variation.

#### 2.5.1. A Multi-Modal Assessment Approach

Research investigating potential welfare effects must include an assessment of more than one of the physical domains within the Five Domains model to assess parallel or indirect effects.

- Physiological indicators: Measure variables such as heart rate, respiratory rate, corticosteroid concentrations, and eye temperature, ensuring appropriate validation for the species and context.
- Behavioural indicators: Record displacement activities, facial expressions, body posture, and critically evaluate candidate indicators of positive, neutral, or negative affect. Video-record all interventions to enable later validation, machine learning analysis, and cross-validation. When using eye thermography, record both eyes to identify differences.
- Physical indicators: Report pressure or force data for the full range (minimum to maximum), along with evidence of tissue damage or lesion assessment.
- Functional indicators: Evaluate range of motion, locomotory behaviour changes, and eating/drinking behaviour.
- Biological lateralisation: Recognise that inherent morphological and neurological asymmetries can manifest as asymmetrical movement patterns that may be mistaken for pathology, e.g., lameness.

#### 2.5.2. Temporal and Technical Considerations

- Acute versus chronic effects: Distinguish between immediate responses and longer-term outcomes.
- Observation durations: Set observation periods long enough to detect effects and consistent with the relevant physiological and/or behavioural processes.
- Context-specific validation of physiological measures: Recognise that relationships between physiological indicators (such as eye temperature, spontaneous blink rate) and welfare states are context-dependent, influenced by stressor type, duration, individual temperament, and environmental factors. Avoid assuming that an indicator validated in one context (e.g., transport stress over hours) is equally valid in another context (e.g., brief equipment trials of seconds).
- Operator effects: Control for inadvertent effects of personnel on animals (e.g., kennel attendants associated with daily feed delivery may increase arousal and positive valence in colony dogs, whereas veterinarians associated with invasive procedures may increase arousal and negative valence in stabled horses).
- Real-world durations: Base observation periods on real-world applications of proposed interventions.
- Equipment and training history effects: Document and account for effects of current and previous equipment use (e.g., noseband tightness constraining oral behaviours, post-inhibitory rebound effects) and training methods that may produce behavioural legacies independent of current pain states.
- Assess with multiple handlers: Where feasible, evaluate animals with different handlers of comparable skill to isolate handler-specific effects (asymmetry, inconsistent contact, centre-of-mass positioning) from animal-specific responses (e.g., pain).
- Post-intervention monitoring: Assess recovery periods after intervention removal to confirm that post-inhibitory rebound effects have returned to baseline, ideally including contemporaneous control animals.

#### 2.5.3. Real-World Relevance

- Ecological validity: Design study conditions to reflect real-world use scenarios.
- Equipment configuration: Replicate the assembly and configurations of test equipment as used in practice (e.g., for horses, include double bridles when studying elite dressage horses, where their use is mandatory).
- Environmental context: Account for the influence of the testing environment on animal and operator behaviour, recognising that it can alter stress responses and confound interpretation.

### 2.6. S—Study Ethics and Standards

Study ethics and standards must feature the 3Rs (replacement, reduction, refinement), welfare endpoints, long-term effects, independence of industry, and risk–benefit analysis. Studies must comply with national, local, and/or institutional laws and/or regulations for prior approval by an appropriately informed animal ethics committee or other such review bodies that have been made fully aware of the impact on animals. In addition, requirements for informed consent should be observed.

#### 2.6.1. Welfare Monitoring

- Welfare management: Implement real-time welfare monitoring with pre-defined endpoints, i.e., intervention (stopping) criteria.
- Welfare assessment: Include written records of assessment of animal health and welfare throughout the studies, preferably using the Five Domains framework [15]. Such assessments may be conducted by any suitably qualified person, preferably independent of the research team.
- Negative affect assessment: Use validated indices to detect negative affects, such as pain and fear, where relevant. These indices should have demonstrated reliability in the species being studied and may be derived from other peer-reviewed studies that used the method or assessed against other behaviour measures.

#### 2.6.2. Risk–Benefit Assessment

- Proportionality: Justify potential risks in relation to the scientific value of the research according to the principle of proportionality. In planning, reduce all welfare harms, even in cases where risks are low. Critically assess whether continued support of traditional practices has merit.
- Research on existing aversive practices: When studies involve deliberate exposure to aversive practices already in widespread use (e.g., physical restraint methods or restrictive equipment), demonstrate that opportunistic or observational approaches are insufficient, and that the research has a credible pathway to improving or replacing current practice. Avoid increasing overall animal exposure to aversive stimuli without proportionate welfare benefit.
- Non-animal alternatives: Identify and justify why alternative methods (e.g., models, simulations) are not sufficient to meet the study objectives.
- Cumulative effect: Evaluate the cumulative effects of multiple research procedures on individual animals over time.
- Welfare endpoints: Establish clear welfare-based endpoints that trigger intervention or study cessation.
- The 3Rs: Apply the principles of replacement, reduction, and refinement. Do not justify causing harm on the grounds that it reflects routine industry practice.

#### 2.6.3. Informed Consent Regarding Potential Harms: What Is Agreed to by Animal Guardians

- Procedures acknowledged and fully explained (animals): These include all procedures to which their animals may be exposed as part of the research study.
- Potential risks acknowledged and fully explained (animals): These include all the potential risks the animals may experience due to their participation.
- Potential risks acknowledged and fully explained (guardians): These include all potential risks to the guardians due to their animal’s participation in the research, including the potential for both physical and psychological harm should their animals be injured or otherwise harmed during the research.
- Researchers answer questions: Opportunities for animal guardians to ask questions and to have them answered by the research team should be required.
- Informed consent to participate: For consent to be informed, all the above practices are required; moreover, consent to participate must be given voluntarily (i.e., completely free from coercion, manipulation, or undue influence from the research team).

### 2.7. S—Species-Relevance and Scientific Rigour

Studies should demonstrate species-relevance and scientific rigour by employing robust methodologies that account for species-typical capacities, motivations, and affective responses, as well as support cross-species applicability and ensure real-world relevance.

#### 2.7.1. Specificity of Behaviour Modification

- Species relevance: Ground study protocols in species-typical behavioural repertoires, cognitive capacities, and sensory modalities, including motivational salience and social dynamics.
- Training contexts: Include protocols specifically designed for training, conditioning, exploration of animal cognition, and behaviour modification research.
- Aversive stimuli: Acknowledge the ubiquity of aversive stimuli in all reports.
- Resource deprivation: Report any deprivation of food, water, or other resources known to be biologically significant using a Five Domains Model assessment [15].
- Learning theory: Specify the theoretical framework of learning and ensure it aligns with the species’ behavioural ecology.
- Cross-species relevance: Note and critically evaluate cross-species applicability where relevant.

#### 2.7.2. Methodology Centred on Welfare

- Acknowledge teleonomic principles: Recognise that welfare indicators are best understood as evolutionary adaptations that enable animals to respond to conditions affecting their biological fitness [16]. Welfare-centred methodology evaluates how procedures engage or constrain the animal’s evolved capacities and motivations in pursuing biologically relevant goals.
- The precautionary principle: Embed precautionary perspectives throughout the research design, implementation, and interpretation of results.
- Acknowledge measurement limitations: When physiological indicators show inconsistent relationships with welfare states in the published literature, researchers must explicitly acknowledge these limitations when interpreting their findings. Absence of a detectable change in a poorly validated or context-inappropriate indicator cannot be interpreted as absence of welfare compromise.
- Welfare assessment and monitoring: Use the Five Domains Model [15] to evaluate outcomes, ensuring that assessment reflects species-specific welfare indicators. Implement real-time welfare monitoring with pre-defined stop criteria to identify and address any emerging welfare risks during procedures.
- Subject selection: Prioritise naïve or minimally habituated animals where appropriate to reduce the confounding effects of prior learning.
- Post-inhibitory rebound: Assess for rebound effects to detect unmet behavioural needs.

#### 2.7.3. Specificity of Equipment and Training Methods (e.g., Detection of Pressure Effects)

- Force and pressure measurements: Use validated instrumentation, such as tensiometers and pressure measurement devices, where appropriate, to quantify mechanical loads applied to tissues.
- Measurement protocols: Provide detailed protocols for recording pressure or force that account for species-specific anatomy and relevant individual variation.
- Equipment fitting: Standardise equipment fitting to allow for replication and comparison across studies.
- Graduated testing: Employ graduated testing of treatments with clearly defined welfare endpoints.
- Sampling sites: Where appropriate, collect data at multiple anatomical sites to capture variation in the mechanical impact of pressure due to variable compliance of underlying tissues.

As noted throughout this commentary, the COMPASS Guidelines complement the foundation of the ARRIVE Guidelines [3] by providing specialised methodological and ethical guidance for behaviour modification research. Similarly, researchers designing behaviour modification studies should also consult the PREPARE Guidelines [4], as recommended above. Together, PREPARE, ARRIVE, and COMPASS form a coherent framework: PREPARE largely deals with the institutional planning stage; ARRIVE provides comprehensive details on ethically based research practice in general; and COMPASS focuses on the specific methodological and ethical considerations for behaviour modification studies. This integrated approach supports the highest standards of scientific rigour, ethical practice, and welfare focus in behaviour modification research.

## 3. Conclusions

These Guidelines represent a framework for conducting rigorous, ethical, and welfare-focused research into the behaviour modification of animals. They have been developed to apply to studies of animal behaviour modification in a wide range of terrestrial mammals and some aquatic species. They are intended to elevate the standards of research in the field of animal training and handling and ensure that scientific inquiry contributes to improved welfare and understanding of animals in the care of humans. Researchers are encouraged to exceed these standards and to contribute to the ongoing refinement of these protocols through their research and experience. The primary goal is to ensure that behaviour modification research provides reliable, ethical, and welfare-focused knowledge that benefits both animals and the humans who work with and care for them. The COMPASS Guidelines should be regarded as “evolving”. Specifically, they should be reviewed and updated as the field continues to evolve and new methodologies and understanding emerge and are validated.

## Data Availability

Not applicable.
